# Assessing dehydration status in dengue patients using urine colourimetry and mobile phone technology

**DOI:** 10.1371/journal.pntd.0008562

**Published:** 2020-09-03

**Authors:** Natalie Chew, Abdul Muhaimin Noor Azhar, Aida Bustam, Mohamad Shafiq Azanan, Crystal Wang, Lucy C. S. Lum

**Affiliations:** 1 Department of Emergency Medicine, University Malaya, Kuala Lumpur, Malaysia; 2 Department of Pediatrics, University Malaya, Kuala Lumpur, Malaysia; International Atomic Energy Agency, AUSTRIA

## Abstract

**Background:**

Dengue is a systemic and dynamic disease with symptoms ranging from undifferentiated fever to dengue shock syndrome. Assessment of patients’ severity of dehydration is integral to appropriate care and management. Urine colour has been shown to have a high correlation with overall assessment of hydration status. This study tests the feasibility of measuring dehydration severity in dengue fever patients by comparing urine colour captured by mobile phone cameras to established laboratory parameters.

**Methodology/Principal findings:**

Photos of urine samples were taken in a customized photo booth, then processed using Adobe Photoshop to index urine colour into the red, green, and blue (RGB) colour space and assigned a unique RGB value. The RGB values were then correlated with patients’ clinical and laboratory hydration indices using Pearson’s correlation and multiple linear regression. There were strong correlations between urine osmolality and the RGB of urine colour, with r = -0.701 (red), r = -0.741 (green), and r = -0.761 (blue) (all p-value <0.05). There were strong correlations between urine specific gravity and the RGB of urine colour, with r = -0.759 (red), r = -0.785 (green), and r = -0.820 (blue) (all p-value <0.05). The blue component had the highest correlations with urine specific gravity and urine osmolality. There were moderate correlations between RGB components and serum urea, at r = -0.338 (red), -0.329 (green), -0.360 (blue). In terms of urine biochemical parameters linked to dehydration, multiple linear regression studies showed that the green colourimetry code was predictive of urine osmolality (β coefficient -0.082, p-value <0.001) while the blue colourimetry code was predictive of urine specific gravity (β coefficient -2,946.255, p-value 0.007).

**Conclusions/Significance:**

Urine colourimetry using mobile phones was highly correlated with the hydration status of dengue patients, making it a potentially useful hydration status tool.

## Author summary

The vast majority of symptomatic dengue infections will result in an uncomplicated disease course. A small proportion will develop severe dengue late in the course of disease, on about day 4 or 5 of illness. In the absence of early prognostic markers to triage dengue patients for early supportive intervention, all suspected dengue cases are followed up for daily assessment, which includes an assessment of dehydration. While the clinical assessment of dehydration is subjective, the passing of dark-coloured urine is an accepted indicator of dehydration. However, visual assessment of urine colour is limited by the varied perception of colour and ambient lighting. An objective method to assess urine colour is to encode it in the RGB model. We used a mobile phone to capture an image of urine samples under standardised conditions and computed the RGB value of each image. Our study demonstrated that RGB urine colourimetry using mobile phones is highly correlated with the hydration status of dengue patients, making it a hydration status tool of great potential.

## Introduction

Dengue is the most rapidly advancing vector-borne disease in humans and a major public health concern in the tropical and subtropical regions of the world. It continues to impose a major burden on healthcare systems. While the vast majority of symptomatic dengue infections manifest in an uncomplicated disease course, severe dengue occurs in a small proportion of infections late in the course of disease, around day 4 to 6 of illness. However, no early prognostic markers have been identified to assist the clinician in triaging dengue patients for early supportive intervention. Thus, all patients with suspected dengue are followed up for daily assessment, which includes a full blood count. Patients with dengue infection are susceptible to dehydration as a consequence of high fever, nausea, vomiting, anorexia and diarrhoea during the febrile phase of 4 to 6 days [1–3]. Fluid intake of more than five glasses during the 24 hours before evaluation by a clinician decreased the risk of hospitalization of dengue fever patients [4]. Hypovolemic shock is often caused by fluid loss into third spaces rather than by bleeding. With prolonged shock, complications such as gastrointestinal bleeding, disseminated intravascular coagulopathy, and multiorgan failure are imminent. Therefore, it is important to accurately assess dehydration severity so that appropriate care and management can be given to mitigate mortality and morbidity.

Plasma volume changes can be estimated from haemoglobin and haematocrit levels, but assessing these parameters is invasive and interpretation may be difficult in the absence of the patient’s baseline value. Serum osmolality is a good marker for critical dehydration situations, but is tightly physiologically regulated and therefore insensitive to mild dehydration, especially in younger populations [5]. In contrast, urinary hydration biomarkers do vary according to fluid intake and losses [6].

It is challenging to determine a patient’s clinical hydration status accurately with just one parameter. Traditional methods of measuring hydration status in patients are subjective, posing a challenge to healthcare workers. As early as the 1970s, clinicians have determined the correlation of clinical dehydration with urine colour, specific gravity (SG), and osmolarity [7]. Darker urine colour is accepted as an indicator of poor hydration status in individuals [8]. A study in Thailand of children admitted for dengue fever and dengue haemorrhagic fever found a high incidence of high urine SG and ketonuria [9]. In a study of critically ill patients without renal disease or impairment, there was a significant correlation between urine colour and the urine-plasma sodium ratio [10].

Hydration dynamic assessment was best accomplished by urine SG, plasma osmolality, and body mass. Urine colour was 81% sensitive but 97% specific [5], and able to track body water loss as effectively as urine osmolality, urine SG, plasma sodium, and plasma osmolality [6, 11]. Urine colour and urine SG were found to be good indicators for hydration assessment in elderly patients and suitable early markers of dehydration [12–14].

A urine colour chart of eight scales developed by Armstrong et al. was found to be valid and easier to use than more routine urinary biomarkers [11]. As urinary indices are sensitive to small changes in hydration status, urine colour is feasible for the detection of early hydration problems. The visual-manual interpretation has limitations surrounding variations in perception of colour, lighting conditions, and timing at which reagents are read [15]. On the other hand, modern cameras and computer displays allow for rapid photo processing in colour balancing and compensation.

There are 3 key challenges to smartphone colorimetric testing–inaccurate image quality due to integrated colour balancing functions, ambient lights, and difficulty in analysing small colour changes [16]. These challenges have been overcome by using a fully-controlled camera function and constructed calibration curves in a process that does not require specially trained personnel and can be accomplished in seconds. A smartphone equipped with a camera can be used for mobile dipstick urinalysis with high precision [17].

A hydration tracker that uses the RGB values of colour, with results further confirmed by urine specific gravity, was developed [18]. The RGB colour model is a reflection of light source that is additive in wavelength, which makes the final colour spectrum a mixture of red, green, and blue light [19]. The RGB colour model is one of the most common ways to encode colour in computing, allowing us to use it as an objective assessment of urine colour.

No previous studies have explored the use of mobile phones in assessing the severity of dehydration in dengue fever. This study aims to further explore the utility of mobile phones in assessing the severity of dengue as it manifests in hydration level, using mobile phone-captured images to correlate urine colour to laboratory parameters.

## Methods

### Recruitment and data collection

We conducted a cross-sectional observational study over six months, from April to September 2016 in the Emergency Department of University Malaya Medical Centre. The study protocol was approved by the Medical Research Ethics Committee of University Malaya Medical Centre (approval number 201512–1942).

All patients between the ages of 12 and 60 years with suspected or confirmed dengue fever were screened. Patients were included if they tested positive for NS-1 Ag or dengue IgM serology, and were excluded if they were pregnant, had hepatic or renal failure or urinary tract infection, or were suffering from malignancy of any kind. History, physical examination, vital signs, blood, and urine samples were taken after a written informed consent was obtained from patients or parents of patients aged <18 years. Urine samples were collected in clear hospital-standard plastic urine bottles. All laboratory assessments were conducted by the Clinical Diagnostic Laboratory of University Malaya Medical Centre.

### RGB Urine colorimetry

**Fig 1:**
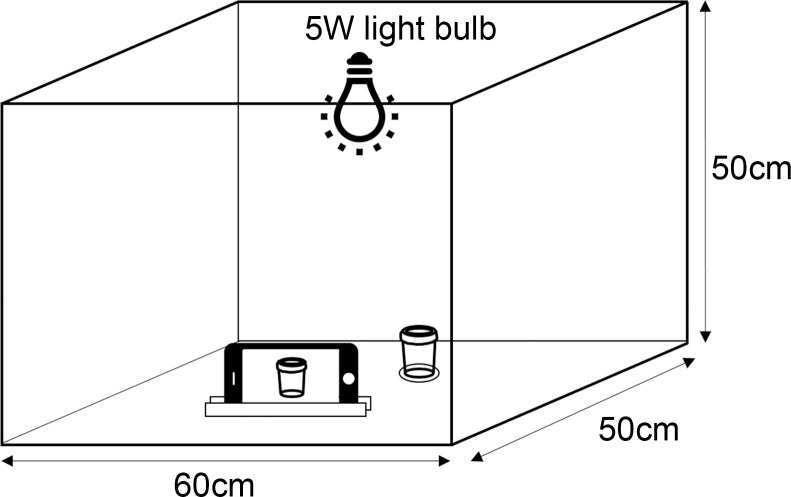
Photo booth setup to capture urine colour. The schematic of the photo booth along with placements of urine bottle and light source.

As shown in Fig 1, a custom-built photo booth with complete enclosure was used to eliminate any effects of environmental light and other ambient factors. The box (60cm x 50cm x 50cm) was made of polystyrene with an internal white background. A 5W ‘cool daylight Philips’ light bulb was placed at the top of the box as the sole light source. A specific site directly under the light bulb with minimal shadow cast was marked for placement of the urine bottle. The box was concealed with the lid to prevent ambient light from creating a colour cast.

Pictures of the urine bottle were taken by Apple iPhone 5S within 2 minutes of the urine samples received from patients to avoid sediments settling at the bottom. An application called Procamera with ISO of 32 and shutter speed of 1/2053s was used to capture high-quality images in.tiff format. A timer set to 10 seconds allowed for the closure of the box lid before image capture to prevent motion artifacts. Adobe Photoshop CC 2015 for Mac was used to analyse the urine images, cropped to leave only the image of the urine without the bottle. An average RGB value for each image was generated using the Average Blur function (Filter > Blur > Average).

The blue component of the RGB urine colourimetry value is categorized according to the Body Hydration Tracker system proposed by Jeanette et al. (2015); values of less than 40, 40 to 100, 100 to 170, and greater than 170 indicate serious dehydration, significant dehydration, minimal dehydration, and well hydration, respectively [18].

### Statistical analysis

The calculation of sample size was based on G*power for Mac version 3.1. The calculated effect size was 0.17 based on the study by Choodum et al. (2014) [20]. The calculated sample size for a study power of 95% and confidence interval of 95% was 77. Including a drop-out rate of 10%, the final calculated sample size was 84.

Data was analyzed using IBM SPSS Statistics SPSS version 20. Descriptive statistics for demographic data, clinical laboratory results, and urinalysis were presented as medians with interquartile ranges. Correlation between hydration status, laboratory parameters, and RGB urine colour values were analysed using Spearman Correlation. Association between categorical parameters were analysed using Mantel-Haenszel statistics. Multiple linear regression was used to predict the biochemical characteristics of urine based on the colourimetry value of RGB.

## Results

A total of 117 patients were enrolled. 15 patients were excluded for negative results of NS-1 Ag and/or IgM tests, and 5 others due to insufficient data. Data from the remaining 97 patients, aged 13 to 60 years, were analysed. Table 1 displays patient and clinical characteristics at time of triage. The majority (58%) of patients exhibited dengue fever with no warning signs, while 41% showed warning signs and only 1 patient progressed to shock. The median systolic blood pressure was 115 mmHg, median diastolic blood pressure 70 mmHg, and median heart rate 90 bpm. Only 5.1% of patients presented with systolic blood pressure less than 100 mmHg, whereas 31.9% had heart rates over 100 bpm. The median pulse pressure was 46 mmHg, with 30% of patients with pulse pressure less than 40 mmHg.

**Table 1:** Patient and Clinical Characteristics at Time of Triage.

	Median [IQR] or no. (%)
Age (years)	25 [21–34]
Day of Fever	4 [4–5]
Systolic BP (mmHg)	115 [109–126]
Diastolic BP (mmHg)	70 [64–79]
Pulse pressure (mmHg)	46 [38.5–52.0]
Heart rate (beats per min)	90 [77–102]
Temperature (^o^C)	37.4 [36.9–38.0]
Male	67 (69)
Dehydration between 5–10%	20 (21)
Not able to drink	16 (17)
Dengue severity	
Dengue fever with no warning signs^a^	56 (58)
Dengue fever with warning signs^b^	40 (41)
Dengue shock^c^	1 (1)
Hospital admission	38 (39)
Intravenous fluid therapy	41 (42)
< 5 ml/kg	24 (25)
5 ml/kg	14 (14)
10 ml/kg	2 (2)
20 ml/kg	1 (1)

Continuous variables are presented as median values with interquartile range (IQR) while categorical data is presented as numbers with percentages.

^a,b,c^ Patients were classified according to Malaysian Clinical Practice Guidelines: Management of Dengue Infection in Adults [21].

Table 2 shows the results of laboratory investigations. The median haematocrit was 45%; 27.8% of patients had haematocrit of less than 40%. Thrombocytopenia, defined as platelet count less than 100 x 10^9^/L, was present in 41.2% of patients, while leucopenia was present in 64.9%. Median values of other blood results fall within normal ranges, except for slightly elevated alanine aminotransferase (ALT) and aspartate aminotransferase (AST). Only 4% of patients had AST greater than 400 IU/L and 1% had ALT greater than 550 IU/L, representing a 10-fold increase in the maximum normal value.

**Table 2:** Laboratory investigation and urine RGB colorimetry code results.

	Median (IQR)	Reference Range
Blood parameters		
White Cell Count (x10^9^/L)	3.5 (2.6–4.4)	4.0–10.0
Haematocrit (%)	45 (40–48)	40–50
Platelet (x10^9^/L)	110 (80–148)	150–400
Sodium (mmol/L)	136 (134–138)	136–145
Urea (mmol/L)	3.7 (3.0–4.4)	3.2–8.2
Creatinine (μmol/L)	78 (66–90)	54–97
Bilirubin (μmol/L)	8 (6–11.5)	< 17
ALT (U/L)	51 (28–125)	10–49
AST (U/L)	70 (45–152)	< 34
Serum Osmolality (mmol/kg)	282 (277–287)	275–295
Urine parameters		
Urine Sodium (mmol/L)	30 (14–72)	
Urine Osmolality (mmol/kg)	525 (273–811)	50–1200
Urine specific gravity	1.016 (1.009–1.022)	1.005–1.030
Urine protein (g/L)	0.25 (0.00–0.25)	
Urine ketone (mmol/L)	0.00 (0.00–1.50)	
Positive urine ketone (n = 38)	5 (5–15)	
Urine urobilinogen (μmol/L)	0 (0.0–17.0)	
Positive urine urobilinogen (n = 35)	17 (17–68)	
Urine bilirubin (μmol/L)	0 (0.0–17)	
Positive urine bilirubin (n = 25)	17 (17–68)	
Urine haemoglobin (/μL)	10 (0–25)	
RGB urine colour value		
Red	242 (210–253)	
Green	202 (148–242)	
Blue	73 (37–159)	

Continuous variables are presented as median with interquartile range (IQR)

Results of the Spearman correlation analysis (Table 3) show strong correlations between urine osmolality and urine SG with the RGB values of urine colour. The blue component showed the highest correlations with urine SG and urine osmolality among all colour components. There were also moderate correlations between RGB components and percentage of dehydration, serum urea and bilirubin, dengue severity, and intravenous fluid therapy.

**Table 3:** Spearman Correlation Analysis between clinical and laboratory parameters with RGB urine colourimetry.

RGB Colourimetry	Red, *r* (p-value)	Green, *r* (p-value)	Blue, *r* (p-value)
Clinical Parameters			
Heart rate	0.072 (0.482)	0.010 (0.924)	0.027 (0.790)
Systolic blood pressure	0.110 (0.284)	0.124 (0.225)	0.102 (0.321)
Diastolic blood pressure	-0.013 (0.900)	0.006 (0.955)	0.005 (0.961)
Pulse pressure	0.175 (0.087)	0.168 (0.101)	0.142 (0.165)
Percentage of dehydration	-0.202* (0.048)	-0.221* (0.030)	-0.222*(0.029)
Laboratory Investigation			
Total white cells	0.005 (0.957)	-0.039 (0.704)	-0.101 (0.325)
Haematocrit	-0.121 (0.238)	-0.105 (0.308)	-0.124 (0.228)
Platelets	0.145 (0.156)	0.119 (0.246)	0.058 (0.575)
Urea	-0.338** (0.001)	-0.329**(0.001)	-0.360**(0.001)
Creatinine	-0.036 (0.729)	-0.038 (0.709)	-0.060 (0.556)
Bilirubin	-0.315**(0.002)	-0.375** (0.000)	-0.371**(0.000)
Serum osmolality	0.006 (0.952)	0.033 (0.749)	-0.001 (0.990)
Urine Analysis			
Urine Osmolality	-0.701** (<0.001)	-0.741**(<0.001)	-0.761**(<0.001)
Urine Sodium	-0.096 (0.349)	-0.120 (0.241)	-0.179 (0.079)
Urine Specific Gravity	-0.759**(<0.001)	-0.785**(<0.001)	-0.820**(<0.001)
Urine Protein	-0.623**(<0.001)	-0.643**(<0.001)	-0.628**(<0.001)
Urine Ketone	-0.623**(<0.001)	-0.606**(<0.001)	-0.583**(<0.001)
Urine Bilirubin	-0.735**(<0.001)	-0.762**(<0.001)	-0.728**(<0.001)
Urine Urobilinogen	-0.584**(<0.001)	-0.644**(<0.001)	-0.617**(<0.001)
Urine Haemoglobin	-0.301**(0.003)	-0.306**(0.002)	-0.280**(0.005)
Clinical			
Dengue Severity	-0.273** (0.007)	-0.274**(0.007)	-0.243*(0.016)
Intravenous fluids therapy	-0.288** (0.004)	-0.278**(0.006)	-0.256*(0.011)

*r* denotes Spearman’s rho coefficient

** denotes p-value <0.01

* denotes p-value <0.05

**Fig 2:**
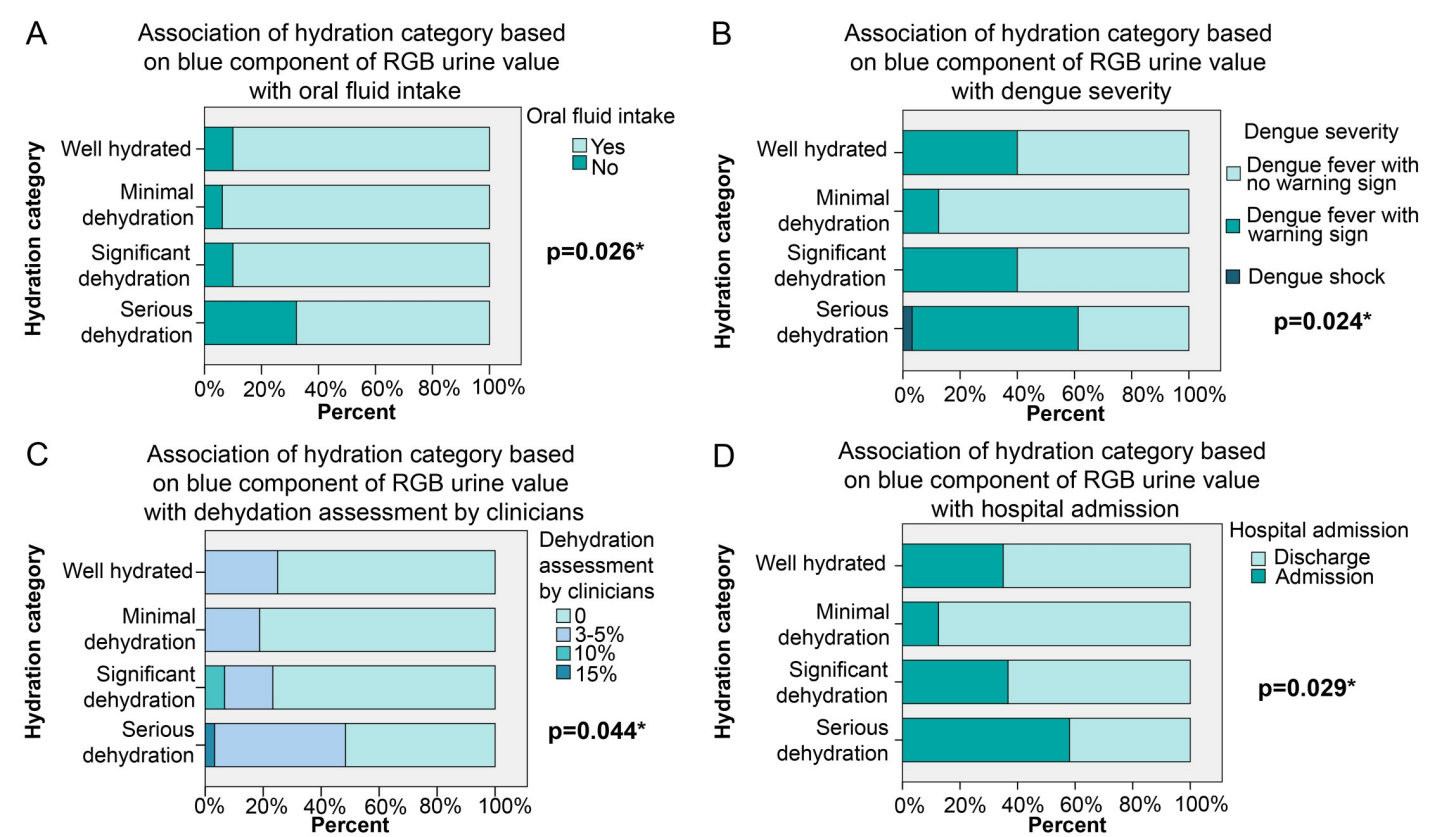
Association of the blue component of RGB urine colour value with dehydration assessment by clinician, dengue severity, oral fluid intake and hospital admission. Hydration category was based on the blue component of RGB urine value according to the Body Hydration Tracker system proposed by Jeanette et al. (2015); a value <40 indicates serious dehydration, 40 to 100 indicates significant dehydration, 100 to 170 indicates minimal dehydration and >170 indicates well hydration [18]. * denotes significant p-value calculated using Mantel-Haenszel statistics.

The blue component of the RGB urine colourimetry was categorized according to the Body Hydration Tracker system proposed by Jeanette et al. (2015) [18]. Based on this scale, 32% of our patients had serious dehydration, 31% of patients had significant dehydration, 17% had minimal dehydration, and only 20% were well hydrated. Hydration categories based on the blue component of RGB urine colorimetry were associated with dehydration assessment made by clinicians, dengue severity, intravenous fluid prescription, and hospital admission (all p<0.05) (Fig 2A–2D).

Results of the multiple linear regression of the three RGB urine colour values against biochemical characteristics of urine analysis are shown in Table 4. The red component had the greatest number of significant associations with urine analysis parameters (sodium, osmolality, protein, urobilinogen, bilirubin and haemoglobin; all p<0.05). Urine analysis parameters linked to dehydration (urine osmolality and urine SG) were also associated with RGB urine colour value. The green colourimetry code best predicted urine osmolality (β coefficient -0.082, p-value <0.001) while the blue colourimetry code best predicted urine SG (β coefficient -2,946.255, p-value 0.007).

**Table 4:** Association of RGB urine colour values with biochemical characteristics of urine analysis using multiple linear regression.

	Unstandardized Coefficients β	Standardized Coefficients Beta	95% Confidence Interval	P-value
Red				
Urine Sodium	0.129	0.264	0.067 to 0.190	<0.001**
Urine Osmolarity	-0.035	-0.413	-0.057 to -0.013	0.002**
Urine Specific Gravity	-171.096	-0.059	-904.547 to 562.356	0.644
Urine Protein	-4.028	-0.146	-7.279 to -0.778	0.016*
Urine Ketone	-0.090	-0.017	-0.727 to 0.546	0.779
Urine Urobilinogen	-0.150	-0.233	-0.233 to -0.067	0.001**
Urine Bilirubin	-0.699	-0.277	-1.092 to -0.306	0.001**
Urine Haemoglobin	-0.054	-0.148	-0.098 to -0.009	0.020*
Green				
Urine Sodium	0.232	0.238	0.123 to 0.341	<0.001**
Urine Osmolarity	-0.082	-0.487	-0.122 to -0.043	<0.001**
Urine Specific Gravity	-389.708	-0.067	-1,692.196 to 912.779	0.554
Urine Protein	-8.849	-0.160	-14.621 to -3.078	0.003**
Urine Ketone	-0.246	-0.023	-1.375 to 0.884	0.667
Urine Urobilinogen	-0.326	-0.2253	-0.474 to -0.178	<0.001**
Urine Bilirubin	-1.247	-0.246	-1.944 to -0.549	0.001**
Urine Haemoglobin	-0.058	-0.080	-0.138 to 0.022	0.151
Blue				
Urine Sodium	0.183	-0.149	0.006 to 0.360	0.042*
Urine Osmolarity	-0.081	-0.379	-0.144 to -0.017	0.014*
Urine Specific Gravity	-2,946.255	-0.403	-5,047.922 to -844.587	0.007**
Urine Protein	-9.601	-0.138	-18.914 to -0.288	0.043*
Urine Ketone	-0.647	-0.049	-2.471 to 1.176	0.482
Urine Urobilinogen	-0.199	-0.123	-0.438 to 0.039	0.101
Urine Bilirubin	-0.083	-0.013	-1.209 to 1.043	0.884
Urine Haemoglobin	-0.020	-0.022	-0.148 to 0.108	0.757

Variables were initially checked for collinearity. All variables have variance inflation factor (VIF) of <10.

** denotes p-value <0.01

* denotes p-value <0.05

## Discussion

Our study evaluated the use of RGB urine colourimetry as a hydration biomarker for older paediatric and adult dengue patients. It shows that RGB values of urine colour were correlated with urinary indices of hydration status in these dengue patients. Urine colourimetry has been applied to measure urinary methamphetamine and degrees of hydration [18, 20]. To our knowledge this is the first study to utilize mobile phone colourimetry techniques to assess hydration status associated with dengue infections.

Dengue patients were selected for our study population because of the significant burden of disease as well as the importance of frequent assessment of hydration status in the clinical management of dengue. Promotion of adequate fluid intake at home could significantly reduce the risk of hospitalization and thus attenuate the economic impact of dengue in countries experiencing epidemics of dengue fever [4]. Implementation of a fluid chart in the management of dengue fever in outpatient clinics could help reduce hospitalization and the need for intravenous fluid [22]. Therefore, an accurate, objective, inexpensive, and non-invasive point-of-care hydration status tool would be a highly valuable adjunct in the promotion of adequate fluid intake in the ambulatory setting during a dengue epidemic.

Multiple studies on athletes and army officers have shown a significant relationship between urine colour, urine SG, and urine osmolality [6]. Although there is no gold standard investigation parameter for dehydration, urine SG is both sensitive and specific as an indicator of hydration status [6]. A previous study has shown increasing plasma osmolality with progressive loss of body water resulting in higher urine SG [23].

Our study found urine osmolality and SG as the parameters that were most strongly correlated with all 3 urine colour parameters. The blue component had the highest correlations with urine SG and urine osmolality. Our findings were consistent with the body hydration tracker system by urine colour, a customized kit equipped with urine test strips, a mobile application, and a handheld refractometer[18].

During acute progressive dehydration, urine colour, urine specific gravity, and urine osmolality can be used interchangeably to track hydration status [6, 11]. Similarly, hydration status biomarkers, notably urine osmolality, urine SG, urine colour, and volume, demonstrate a high degree of collinearity across a broad range of values [24]. Our study showed a comparably strong correlation between urine osmolality and urine specific gravity, with r = 0.922 (Supplementary S1 Table).

The RGB components of urine colour in our study were not correlated with vital signs, serum osmolality, and haematocrit. This is consistent with previous studies that showed no significant relationship between dehydration status and blood biochemistry or between urine colour, urine osmolality, and urine specific gravity and serum osmolality, serum sodium, and total plasma protein [6, 11, 25]. Other hydration biomarker studies demonstrated a significant association between total fluid intake and urinary biomarkers, but not plasma osmolality [14, 24]. Urinary biomarkers included 24-hour urine osmolality, colour, urine SG, volume, and solute concentrations. Perrier et al. also demonstrated that both urine colour and urine SG may be used as surrogates for urine osmolality to identify individuals above or below the 500 mOsm/kg target of euhydration [24]. Armstrong et al. showed that urine colour tracked changes in body water as effectively as urine osmolality, SG, and volume during various stages of dehydration [11]. Several other studies have demonstrated significant correlations between urine colour, urine SG, and osmolality as indicators of hydration status [6, 11–14, 24, 26].

In our study, the RGB urine colour values were significantly associated with the inability to drink and weakly correlated with blood urea levels, in accordance with conventional indicators of dehydration. Interestingly, our study also demonstrated a weak correlation between the clinical dehydration status assessed by clinician and RGB urine colourimetry. A study of a 3-day voluntary water restriction resulted in increases in urine osmolality, urine colour, and urine SG [27], a finding similar to that of 24-hour controlled dehydration [14]. Hydration category based on the blue component of RGB urine colourimetry as proposed by Jeanette et al. (18) showed that subjects with poor oral intake were the ones with serious dehydration, while the patient with dengue shock had serious dehydration and those with warning signs had significant or serious dehydration. Except for a rapid decrease in platelet count, the other warning signs of severe dengue such as severe abdominal pain, persistent vomiting, severe lethargy, fluid accumulation, and a rising haematocrit could be related to dehydration. Seven of the 8 subjects who were well-hydrated (based on the blue component of urine colourimetry) but classified as having dengue with warning signs had platelet counts less than 100 x10^9^/L, while the 8^th^ subject had an elevated creatine kinase level at the time of enrolment; they were thus admitted for hospital management.

### Limitations of study

The collection of urine samples was done at the time of patient presentation in the emergency department, with no standardized timing for collection and no early morning urine samples collected. We were not able to collect 24-hour urine samples as most patients voided before coming to the hospital. However, a study by Stover et al. concluded that the time of the day when the urine is collected does not affect the urine SG [28]. In a study of 82 healthy volunteers, first morning urine collected was not correlated with concurrent fluid intake [24]. Although we did not quantify fluid intake in the previous 24 hours, reporting by patients of their inability to take oral fluid was associated with the blue component of RGB urine colourimetry. Due to time constraints we were not able to enrol a larger sample size. Another limitation of our study was that the effect of diet and medications on urine colour was not controlled for and that urine colour changes in our patients were attributed to their dehydration status. Future studies should control for the effect of certain foods and medications on urine colourimetry.

## Conclusion

Our study demonstrated that RGB urine colourimetry using mobile phones is highly correlated with the hydration status of dengue patients, making it a hydration status tool of great potential. As this method is simple, fast, inexpensive, and non-invasive, it can be applied not only for primary healthcare, but for affordable home monitoring as well. With this tool, a quick assessment of hydration status is easily obtained to aid in the management of dengue patients by medical personnel and patients themselves. This study demonstrated that RGB information obtained with a generic camera phone (iPhone 5S, Apple Inc. 2014) which featured an 8 megapixel camera, fixed aperture of f/2.2, 1/3 inch sensor format, 29 mm (standard), and pixel pitch of 1.5 microns could be used for urine colorimetry. Different camera sensors may produce different RGB profiles, but colour calibration can be performed to obtain accurate RGB information [29].

With the high availability and accessibility of smartphones even in difficult-to-access rural areas, a hydration tracker application is a valuable tool for patients and medical personnel. This unique point-of-care tool can be made easily accessible as a downloadable application for those who own a smartphone, and can be linked to infectious disease physicians in major tertiary centres for better care.

Future urine colourimetry studies can be designed with a longitudinal element to track changes in RBG values of urine colour as patients are rehydrated during treatment. Other possible hydration correlations with RGB urine colour values to investigate include intravenous vena cava diameter, left ventricular outflow tract velocity time integral, and central venous pressure.

## Supporting information

S1 Table Spearman correlation analysis between urine biochemical parameters. (DOCX)
