# Supplementary material for: Assessing dehydration status in dengue patients using urine colourimetry and mobile phone technology
Source: PLoS Negl Trop Dis. 2020 Sep 3;14(9):e0008562. doi: 10.1371/journal.pntd.0008562 (PMC7470395; doi:10.1371/journal.pntd.0008562)
Supplement: S1 Table — (DOCX) [file pntd.0008562.s001.docx]

**Supporting Information**

**S1 Table. Spearman correlation analysis between urine biochemical parameters**.

|  | **Sodium** | **Osmolarity** | **Specific gravity** | **Protein** | **Ketone** | **Urobilinogen** | **Bilirubin** |
| --- | --- | --- | --- | --- | --- | --- | --- |
| **Osmolarity** | **0.529****  **(<0.001)** |  |  |  |  |  |  |
| **Specific gravity** | **0.450****  **(<0.001)** | **0.922****  **(<0.001)** |  |  |  |  |  |
| **Protein** | -0.066  (0.521) | **0.483****  **(<0.001)** | **0.558****  **(<0.001)** |  |  |  |  |
| **Ketone** | 0.035  (0.737) | **0.527****  **(<0.001)** | **0.559****  **(<0.001)** | **0.412****  **(<0.001)** |  |  |  |
| **Urobilinogen** | 0.145  (0.156) | **0.508****  **(<0.001)** | **0.532****  **(<0.001)** | **0.375****  **(<0.001)** | **0.349****  **(<0.001)** |  |  |
| **Bilirubin** | 0.115  (0.263) | **0.596****  **(<0.001)** | **0.626****  **(<0.001)** | **0.543****  **(<0.001)** | **0.504****  **(<0.001)** | **0.664****  **(<0.001)** |  |
| **Haemoglobin** | **-0.261***  **(0.010)** | 0.158  (0.123) | **0.266***  **(0.008)** | **0.537****  **(<0.001)** | **0.237***  **(0.019)** | 0.087  (0.395) | 0.193  (0.058) |

Associations were presented as Spearman’s rho coefficient (p-value)

^**^ denotes p-value <0.01

^*^ denotes p-value <0.05
